# Prospective randomized analysis of operating time and safety of laparoscopic pyeloplasty with or without TriSect rapide® in adult patients with ureteropelvic junction obstruction

**DOI:** 10.1186/s12894-026-02257-4

**Published:** 2026-07-15

**Authors:** Bastian Hummel, Gereon Schaelte, Bogdan Baleanu-Curaj, Stefan Istin, Tibor Szarvas, David Schroeders, Boris Hadaschik, Christian Niedworok

**Affiliations:** 1 Department of Urology, University Hospital Essen, University of Duisburg- Essen, Hufelandstr. 52, Essen, 45147 Germany; 2 Department of Urology, Hermann-Josef-Hospital Erkelenz, Erkelenz, Germany; 3 Department of Anesthesiology, University Hospital RWTH Aachen, Aachen, Germany; 4 Department of Anesthesiology, Intensive Care & Emergency Medicine, Hermann-Josef-Hospital Erkelenz, Erkelenz, Germany; 5 Department of Urology, Semmelweis University Budapest, Budapest, Hungary

**Keywords:** Pyeloplasty, Laparoscopic pyeloplasty, Ureteropelvic junction obstruction, Anderson-Hynes, Culp-Deweerd, Surgical tool, Safety, Quality of life, Prospective randomized analysis

## Abstract

**Background:**

Multifunctional instruments like the TriSect rapide^®^ (Erbe Elektromedizin GmbH, Tübingen, Germany) aim to simplify complex surgical procedures by integrating dissection, cutting, and coagulation in a single device. This study aims to evaluate its clinical utility in laparoscopic pyeloplasty. Specifically, we sought to determine whether the use of the TriSect rapide^®^ could significantly reduce the preparatory phase of laparoscopic pyeloplasty by 30% or more compared to conventional instruments.

**Methods:**

In this prospective, randomized trial, 22 patients undergoing laparoscopic pyeloplasty for ureteropelvic junction (UPJ) obstruction were allocated to receive surgery using either the TriSect rapide^®^ instrument set or conventional instruments. The primary endpoint was the duration of the operation (preparation time). Secondary outcomes were perioperative complications and reported quality of life.

**Results:**

The median follow-up was 46 weeks. No significant differences were observed between groups regarding demographic factors, renal function, or intra- and perioperative complications. The intervention group demonstrated a significantly shorter preparation time (83 ± 29.8 vs. 122 ± 32.5 min, *p* = 0.009), resulting in a reduced total operative time (170 ± 32.0 vs. 211 ± 41.6 min, *p* = 0.017) and shorter anesthesia duration (221 ± 41.0 vs. 259 ± 41.5 min, *p* = 0.046). Reconstruction time did not differ significantly (87 ± 14.6 vs. 89 ± 11.1 min, *p* = 0.639).

Complication rates according to the Clavien–Dindo classification were comparable between groups (*p* = 0.935).

Quality-of-life analysis using the RAND SF-36 questionnaire revealed no between-group differences at either time point, although both groups showed significant postoperative improvement compared with baseline (*p* < 0.001).

**Conclusion:**

The use of the TriSect rapide® multifunctional device in laparoscopic pyeloplasty is safe and effective for preparation, dissection and incision. Its application significantly reduces preparation time by approximately 32% without increasing perioperative complications or affecting postoperative quality of life. These findings support the clinical value of multifunctional instruments in improving procedural efficiency while maintaining safety in reconstructive urologic surgery.

**Trial registration:**

In compliance with international guidelines for clinical trial transparency, this study was retrospectively registered in the ISRCTN registry (registration number: ISRCTN15328718) on August 7, 2025.

## Introduction

Multifunctional instruments play an evolving role in surgical practice. Especially devices combining key operative functions — dissection, cutting, and coagulation— reduce the need for instrument exchanges and streamline complex procedures. These innovations are designed to simplify complex surgical procedures by enhancing efficiency and operative time [1]. The aim of this study was to assess the clinical utility of the TriSect rapide→, a novel multifunctional device in laparoscopic urologic surgery. The study focused particularly on laparoscopic reconstructive pyeloplasty, which remains a technically demanding surgical procedure. Pyeloplasty is a standard procedure to correct ureteropelvic junction (UPJ) obstruction, a condition characterized by impaired urine flow due to a narrowing or blockage between the renal pelvis and the ureter, potentially leading to hydronephrosis and kidney damage. While traditionally performed via open surgery, pyeloplasty is increasingly being carried out laparoscopically due to advantages such as reduced postoperative pain, shorter hospital stays, and faster recovery [2, 3]. However, laparoscopic pyeloplasty remains technically challenging, requiring precise dissection and suturing in a confined abdominal working space. Moreover, the frequent need to switch between instruments increases procedural complexity, prolongs operative time, and may elevate the risk of inadvertent injury [4]. 

The TriSect rapide^®^ is a multifunctional surgical device designed to combine dissection, cutting, and coagulation in a single instrument. This integration reduces the need for frequent instrument exchanges, thereby simplifying laparoscopic procedures such as pyeloplasty. The primary aim in using such devices is to improve surgical efficiency by allowing seamless transitions between functions, which may shorten operative time and enhance precision [1]. In addition to technical performance, the device may support a smoother surgical workflow by minimizing interruptions and reducing cognitive load on the surgeon. Its integrated coagulation capability may also provide improved hemostatic control, particularly in highly vascularized tissues, thus maintaining optimal visibility during delicate reconstructive procedures [5]. Beyond these technical aspects, operative duration itself has clinical relevance, as prolonged laparoscopic procedures have been associated with increased anesthesia exposure, postoperative morbidity, and longer hospital stay [6–8]. Consequently, improving surgical workflow and reducing operative time without compromising safety are important goals in minimally invasive reconstructive urology. Devices that integrate multiple functions may therefore contribute not only to greater technical efficiency but also to enhanced perioperative safety and overall surgical quality.

Hence, while the present study focuses on laparoscopic pyeloplasty, the TriSect rapide^®^ shows potential for broader use in various urological surgeries involving the kidney, bladder or prostate. By enabling multiple functions with one tool, it may support or enhance the surgeon`s manual skills and contribute to the standardization of techniques across procedures—ultimately improving surgical outcomes and quality of care.

In this prospective, randomized trial, we evaluated whether the TriSect rapide^®^ could enhance surgical performance by reducing operative time without adversely affecting patient outcomes, quality of life, or the rate of perioperative complications, compared to standard laparoscopic instruments. We specifically hypothesized that the use of the TriSect rapide^®^ would result in a reduction of at least 30% in the operative time required for the preparatory phase of laparoscopic pyeloplasty compared with conventional laparoscopic instrumentation.

## Patients and methods

### Inclusion criteria

Inclusion criteria for the present study were confirmed UPJ obstruction, patient`s age between 18 and 80 years, ASA-performance status 1–3, eligibility for surgical intervention, life expectancy ≥12 months, ability to provide written informed consent and willingness to comply with scheduled follow-up visits.

### Exclusion criteria

Patients who were unable or unwilling to provide informed consent, pregnant patients, patients under the age of 18 or over 80, and those who were either unfit or unwilling to undergo surgery were excluded from participation in the study. Further exclusion criteria were prior surgery involving the retroperitoneum, ureter or kidney, anticipated noncompliance with follow-up requirements, chronic substance abuse, insurmountable language barriers and concurrent participation in another clinical study.

### Study procedure and follow-up

The study protocol was developed in compliance with the CONSORT 2010 statement for the reporting of randomized controlled trials [9]. Between September 2023 and May 2025, 478 patients with symptomatic hydronephrosis were identified in our department. Symptomatic hydronephrosis was defined as the presence of pain, recurrent urinary tract infections, or impaired renal function parameters in patients with sonographically confirmed dilatation of the renal pelvicalyceal system [10]. 

**Fig. 1:**
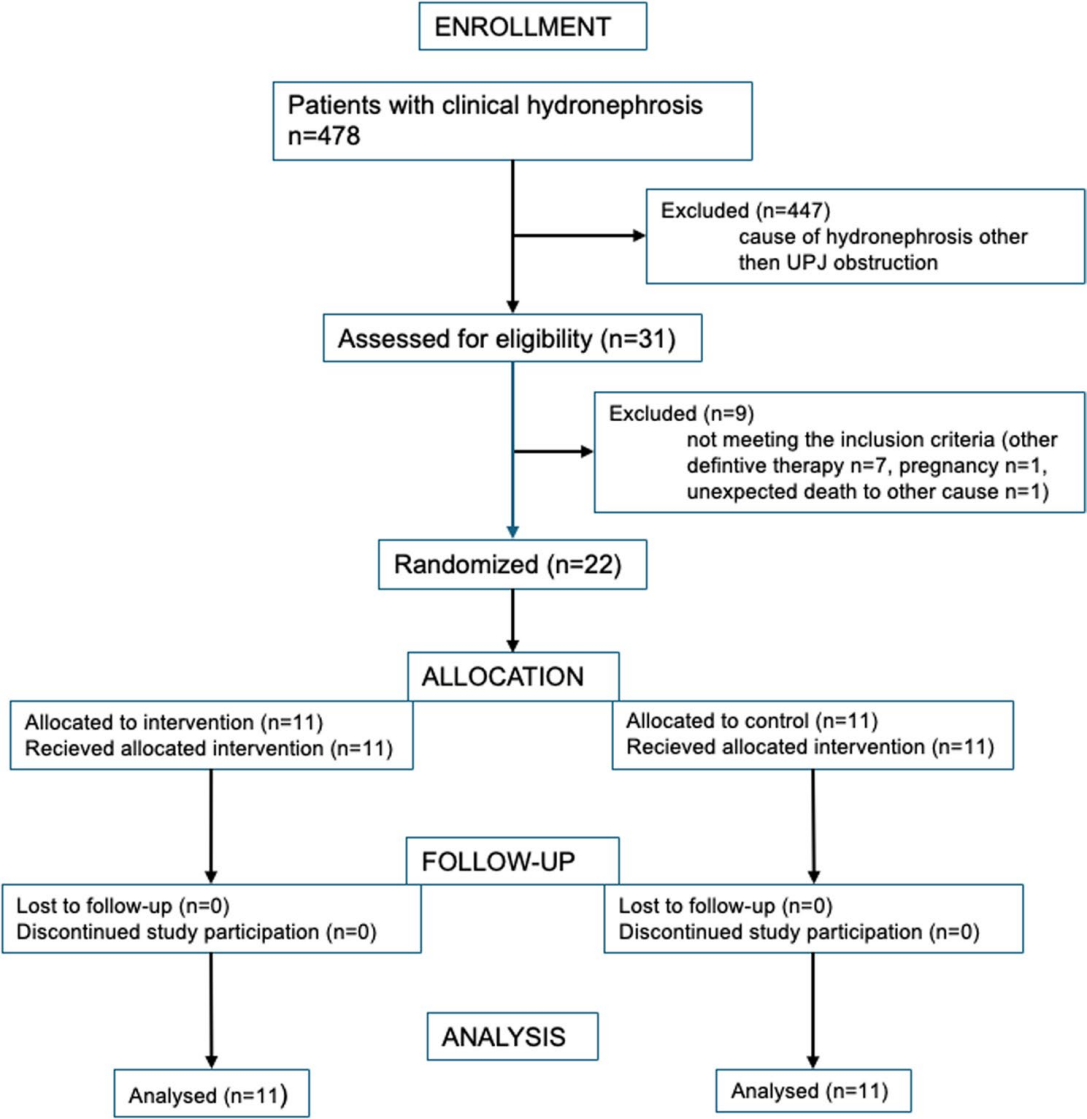
Flowchart in accordance with the CONSORT 2010 guidelines showing patient enrollment, randomization, intervention allocation, follow-up, and inclusion in the final analysis

Initial diagnostic workup included ultrasound and laboratory testing, followed by CT imaging and renal scintigraphy. In patients with CT-confirmed ureteropelvic junction obstruction, additional endoscopic evaluation was performed to identify the underlying cause of the urinary transport disorder [11]. In total, 31 patients with ureteropelvic junction (UPJ) obstruction were identified. In all cases, initial symptomatic management was achieved by placement of a double-J stent [12]. In nine patients, double-J stent insertion remained the temporary definitive therapy due to advanced age, comorbidities, refusal of surgery (e.g., pregnancy), or unexpected death. Ultimately, 22 patients met the inclusion criteria and were randomized to either the intervention group (TriSect rapide^®^) or the control group. Participants were randomly assigned (1:1) to the intervention or control group using a computer-generated randomization sequence with a block size of 4. Allocation was concealed using sequentially numbered, opaque, sealed envelopes prepared by an independent individual (Fig. 1). Due to the visible nature of the surgical instruments used in the operating field, neither the operating surgeons nor the patients were blinded to group allocation.

Follow-up was performed from baseline to July 2025. All patients underwent a standardized follow-up protocol consisting of an initial and a postoperative visit, a six-week follow-up at which the double-J stent was removed, and a final evaluation twelve weeks postoperatively. All patients were contacted in July 2025 to assess the occurrence of any complications since their last follow-up.

### Surgical techniques

**Fig. 2:**
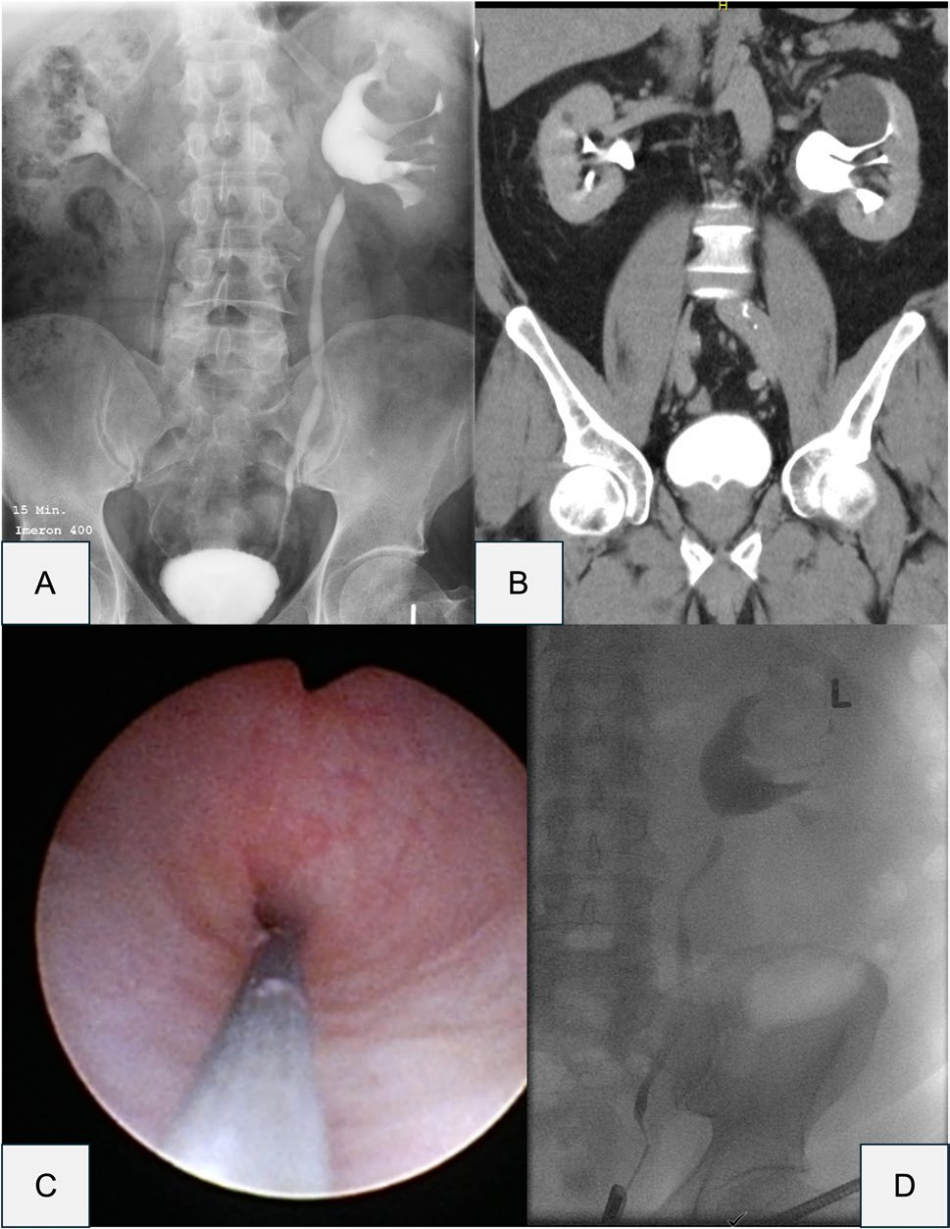
Imaging of a 62-year-old patient with symptomatic ureteropelvic junction (UPJ) obstruction. **A** Intravenous pyelogram showing delayed contrast excretion from the left renal pelvis and typical appearance of UPJ obstruction. **B** Contrast-enhanced CT confirming the diagnosis and demonstrating a non-obstructing upper-pole renal cyst. **C** Endoscopic view of the stenosis with exclusion of tumor or stone. **D** Retrograde pyelography illustrating the classic UPJ obstruction pattern

All patients underwent ureterorenoscopy (URS) with guidewire-assisted placement of a double-J stent under direct visual control [11]. URS was performed under general anesthesia using flexible or semirigid ureteroscopes. Retrograde urography was conducted to assess obstruction, exact localization, the extend of renal pelvic ectasia and to exclude concomitant pathology. Finally, a double-J stent was inserted, none of which remained in situ for longer than six months prior to the subsequent pyeloplasty (Fig. 2). After diagnostic evaluation and establishment of the surgical indication, no stent removal was performed before pyeloplasty.

Laparoscopic pyeloplasty was performed with the patient in a 70° lateral position using four trocars. All procedures were performed by the same surgical team, consisting of one main and one assisting surgeon who alternated roles, resulting in a 1:1 distribution of operations. The surgical staff remained constant throughout the study, and all team members had at least five years of laparoscopic experience. The procedure was divided into a preparatory and a reconstructive phase. Operative time was documented at multiple predefined points: following trocar placement, upon completion of the preparatory phase, after completion of the reconstructive phase, at skin closure, and at the end of anesthesia. The preparatory phase was defined as the time interval from skin incision and trocar placement to completion of the skin suture, excluding all reconstructive steps. This phase included trocar positioning, exposure of the surgical field, dissection of the ureteropelvic junction, and mobilization of the renal pelvis. The reconstructive phase was defined as the period from the initial incision of the renal pelvis to completion of the final suture of the ureteropelvic anastomosis. The exchange of the double-J stent, which was performed prior to completion of the anastomosis, was considered part of the reconstructive phase. Total anesthesia time was measured from administration of the first anesthetic agent until extubation.

**Fig. 3:**
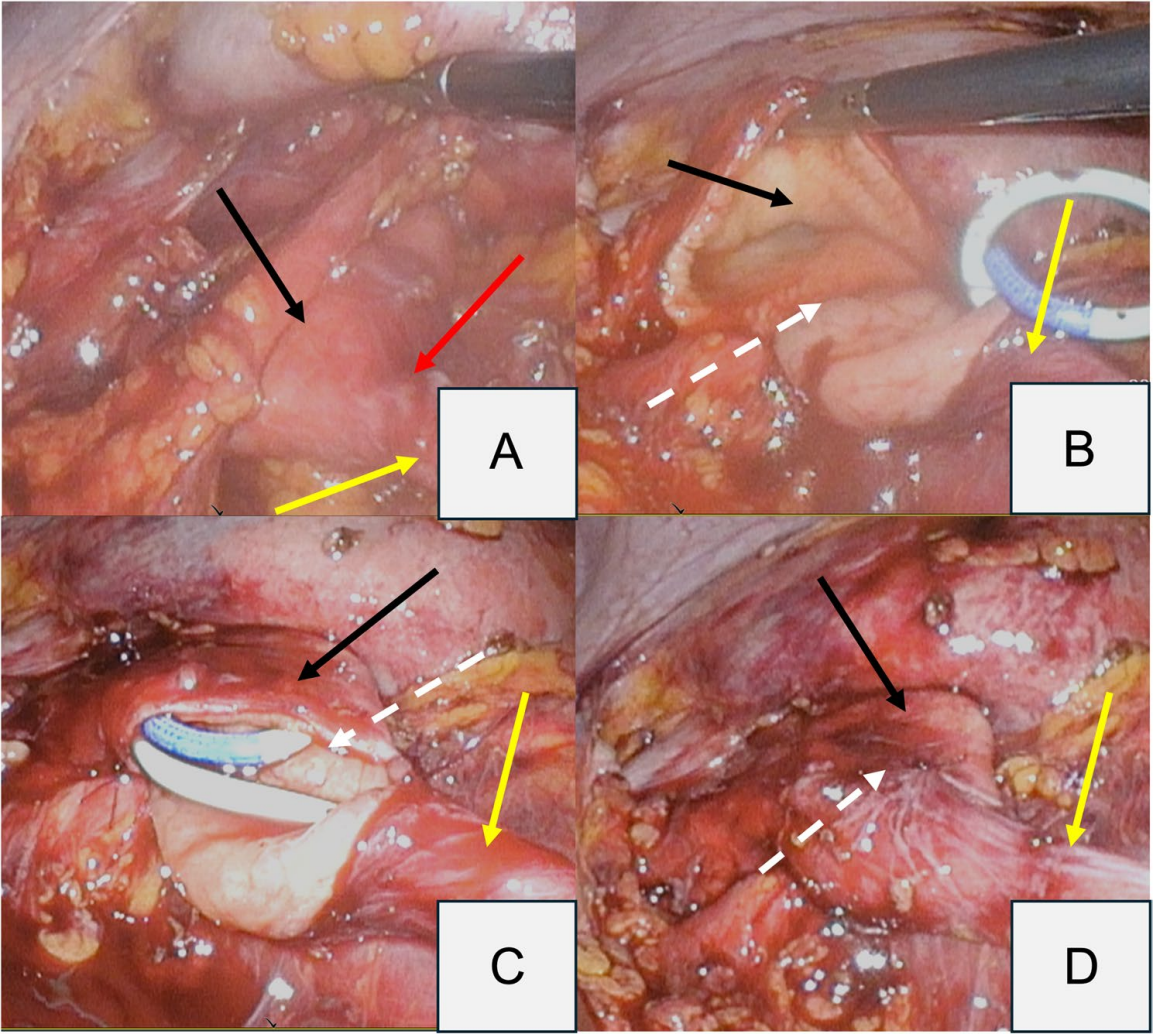
Intraoperative findings in a 48-year-old female patient with ureteropelvic junction (UPJ) obstruction. **A** Dilated renal pelvis, UPJ stenosis, and proximal ureter after dissection using the TriSect rapide→ instrument. **B** Opened and volume-reduced renal pelvis and spatulated proximal ureter with an in situ double-J stent. **C** Pyeloureteral junction after completion of the posterior anastomosis. **D** Final operative view after completion of the full pyeloureteral anastomosis. Arrow legend: Yellow = ureter; black = renal pelvis; red = UPJ obstruction; dashed white = anastomosis

In the intervention group, the TriSect rapide^®^ device was employed for tissue preparation, dissection, and coagulation. Reconstruction and anastomosis of the ureteropelvic junction was performed using the Anderson-Hynes or Culp-DeWeerd technique by Monocryl 4 − 0 sutures. In the control group, eight patients underwent the Anderson–Hynes and three the Culp–DeWeerd procedure, whereas in the intervention group, nine and two patients, respectively, were treated with these techniques. Renal pelvis closure was performed using a single-layer, continuous, full-thickness suture technique. Before renal pelvis closure, the pre-existing double-J stent was exchanged intraoperatively through one of the laparoscopic trocars, ensuring that each patient had a newly placed stent at the end of the procedure. The pyeloureteral anastomosis was constructed with interrupted sutures in both groups (Fig. 3). The double-J stents placed intraoperatively were removed six weeks postoperatively [13]. 

### Evaluation of complications and comorbidities

Clinical and pathological data were prospectively collected from departmental patient charts, medical records, and the treating office-based urologists. Postoperative complications were assessed according to the Clavien-Dindo classification, with the highest grade recorded during the postoperative course considered for analysis [14]. Patients` comorbidity status and general physical performance were assessed using the Charlson Comorbidity Index (CCI) and the ASA Physical Status Classification System [15]. Polypharmacy was defined as the intake of four or more medications. This corresponds to a definition of moderate multimedication, as commonly used in the literature and defined by the World Health Organization (WHO) [16, 17]. 

### Quality of life

Quality of life was evaluated using the RAND SF-36 survey, (SF-36, Version 1.0), a validated questionnaire that assesses the patient-related quality of life and symptom burden in 36 items [18]. It covers physical functioning, pain, role limitations due to physical health problems, role limitations due to personal or emotional problems, emotional well-being, social functioning, energy/fatigue, and general health. Each item scores from 0 to 100, low scores correspond to poorer quality of life. The questionnaire was handed out to each patient at the first time of in-house consultation and twelve weeks after completion of treatment.

### Statistical analysis

Data are presented as medians ± standard error of the mean (SEM). Statistical significance was defined as *p* < 0.05. Non-normally distributed data were analyzed using the non-parametric two-tailed Wilcoxon signed-rank test for paired samples, or the Mann–Whitney U test for unpaired comparisons, as appropriate. For normally distributed variables with unequal variances, Welch’s t-test was applied. Group differences in categorical variables were assessed using the Chi-square test, excluding categories with zero occurrences across all groups.

Prior to patient enrollment, a sample size calculation was performed using the software G*Power (Version 3.1.9.6) [19]. Parameters were based on the expected preparation time during surgery, assuming a mean of 120 min in the control group, a standard deviation of 30 min, and an anticipated 30% reduction in the intervention group. Using a two-sided alpha level of 0.05, a power of 0.8, and an allocation ratio of 1:1, a total of 22 patients (11 per group) was calculated as sufficient to detect the expected effect.

The primary endpoint of this study was the duration of the preparatory phase of surgery. Secondary endpoints included postoperative quality of life and perioperative complications, classified according to the Clavien–Dindo system.

## Results

### Study population and safety

A total of 22 patients were randomized in a 1:1 ratio to the intervention or control group, with no dropouts following randomization. The median follow-up time was 46 weeks and did not differ significantly between the groups (control: 45 ± 16.4 vs. intervention: 41.0 ± 32.9 weeks; *p* = 0.743).

No statistically significant differences were observed between groups in terms of demographic or clinical baseline characteristics. Patients in the control group had a median age of 59 years compared to 47 years in the intervention group (*p* = 0.055), with similar body weights (75 vs. 80 kg; *p* = 0.887), heights (165 vs. 173 cm; *p* = 0.304), and BMI (25.7 vs. 24.8 kg/m²; *p* = 0.126).

Renal function was comparable, with split renal function at 47% vs. 44% (*p* = 0.693) and glomerular filtration rates of 75 vs. 80 ml/min (*p* = 0.685) in the control and intervention groups, respectively.

Perioperative parameters also showed no significant differences. Median hospital stay was 5 days in both groups (*p* = 0.929), the intraoperatively collected fluid volume was 280 vs. 150 ml (*p* = 0.173), and hemoglobin difference between pre- and postoperative values was identical at 1.9 g/dl (*p* = 0.536) (Table 1).

**Table 1:** Baseline characteristics of the study population

		Control			Intervention			
	Parameter	Median (IQR (25–75%))	Mean (±SD)	Range (Min-Max)	Median (IQR (25–75%))	Mean (±SD)	Range (Min-Max)	*p*-value (95% CI)
A	Age (Years)	59 (53.5–64.0)	58 (10.2)	34–71	47 (32.0–56.0)	46 (16.8)	22–77	0.055 (-0.273-24.455)
	Weight (kg)	75 (68.0–85.0)	77 (13.9)	58–103	80 (65.5–89.0)	76 (15.6)	50–98	0.887 (-12.216-14.035)
	Height (cm)	165 (164.5–179.0)	172 (11.8)	158–191	173 (170.0-185.5)	177 (11.7)	156–197	0.304 (-15.688-5.143)
	BMI (kg/m^2^)	25.7 (24.4.-27.3)	25.8 (2.1)	22.9–29.5	24.9 (23.1–26.0)	24.0 (3.1)	16.7–27.4	0.126 (-0.556-4.194)
	Renal Function (% of Overall Function)	47 (41.5–51.0)	46 (8.0)	28–57	44 (37.5–47.0)	44 (13.6)	21–77	0.693 (-8.022-11.840)
	Glomerular Filtration Rate (ml/Min)	75 (52.5–83.0)	69 (20.6)	40–102	80 (56.1–76.7)	66 (20.3)	23–97	0.685 (-14.571-21.733)
B	Hospital Stay (Days)	5 (5.0–7.0)	6 (2.2)	3–11	5 (4.0–7.0)	6 (2.5)	3–11	0.929 (-2.004-2.185)
	Volume of Fluid collected (ml)	280 (165–325)	263 (138)	80–550	150 (65–225)	172 (163)	20–600	0.173 (-43.538-225.54)
	Hb Difference (g/dl)	1.9 (1.05–2.45)	1.9 (1.1)	0.5–4.1	1.9 (0.35–2.45)	1.6 (1.3)	0-3.4	0.536 (-0.736-1.372)

Comparison of preoperative [A] and perioperative [B] parameters between the intervention and control groups. No statistically significant differences were observed in any of the analyzed variables. Data are presented as median (IQR 25–75%) and mean (± standard deviation (SD)), range (min–max), and p-value (95% confidence intervals (95% CI)). Comparisons between groups were performed using independent-samples t-tests for continuous variables. Equality of variances was assessed using Levene’s test. Where homogeneity of variance was confirmed (*p* > 0.05), results were reported under the assumption of equal variances; otherwise, the Welch correction was applied

No statistically significant differences were observed between the groups regarding intra- and perioperative complications (Clavien-Dindo classification, *p* = 0.935), ASA physical status (*p* = 0.281), comorbidity burden as assessed by the Charlson Comorbidity Index (CCI, *p* = 0.344), or medication complexity (*p* = 0.536) (Table 2). Importantly, the intervention group did not experience an increased rate of wound healing disorders or postoperative complications. In both groups, one patient each developed double-J stent occlusion requiring secondary ureterorenoscopy for stent replacement (Clavien–Dindo III). Grade II complications consisted of urinary tract infections treated with antibiotics, occurring in three patients per group. Grade I complications included transient postoperative pain requiring intravenous analgesic therapy in five patients in the control group and six patients in the intervention group.

**Table 2:** Preoperative risk assessment and postoperative outcomes

	Parameter	Category, Grade	Control (n=11)	Intervention (n=11)	*p*-value
A	ASA-Classification	I	3	1	0.281
		II	7	8	
		III	1	2	
		IV-V	0	0	
	Charlson Comorbidity Index (Scores)	0	1	3	0.344
		1	3	5	
		2	3	0	
		3	2	0	
		4	2	1	
		5	0	1	
		6	0	1	
	Medication (number of regular drugs)	0	3	6	0.536
		1	4	1	
		2	2	2	
		3	2	1	
		≥4	0	1	
B	Complications (Clavien-Dindo Grade)	0	2	1	0.935
		I	5	6	
		II	3	3	
		III	1	1	
		IV-V	0	0	

Comparison of the two cohorts regarding preoperative parameters—ASA classification, Charlson Comorbidity Index (CCI), and number of regular medications [A]—as well as postoperative complications according to the Clavien–Dindo classification [B]. Data are presented as absolute numbers of patients (*n*). Group comparisons were performed using Fisher’s exact test (two-tailed). No statistically significant differences were found between the control and intervention groups in any of the analyzed parameters

### Duration of surgery

Preparation time was significantly shorter in the intervention group compared to the control group (83 vs. 122 min, *p* = 0.009, approximately 32% time reduction). This reduction resulted in a shorter total operative time (170 vs. 211 min, *p* = 0.017) and a decreased duration of anesthesia (221 vs. 259 min, *p* = 0.046). Stratified analyses by operating surgeon, BMI, and patient height revealed no significant differences in operative times. Similarly, for the reconstructive phase of the procedure, no significant time differences were observed between groups when stratified by surgical device, BMI, surgeon, or patient height (Table 3).

**Table 3:** Intraoperative time parameters including total surgery time, anesthesia time, preparation time, and reconstruction time

Parameter	Stratification	*n* (per group)	Mean ±SD (range (min-max))	Median	*p*-value (95% CI)
Total Surgery Time (Min.)	Device	11/11	Control 211 ± 41.6 (146–297) Intervention 170 ± 32.0 (114–220)	211 165	0.017 (8.200-74.164)
	BMI	9/13	Normal 183 ± 40.0 (114–243) Overweight 195 ± 44.1 (139–294)	175 188	0.534 (-50.098-26.765)
	Height	12/10	< 171 cm 202 ± 45.6 (146–294) ≥171 cm 176 ± 33.7 (114–220)	205 170	0.147 (-10.049-62.582)
	Surgeon	11/11	A 185 ± 38.3 (139–245) B 195 ± 51.4 (114–294)	169 205	0.578 (-48.139-27.593)
Preparation Time (Min.)	Device	11/11	Control 122 ± 22.5 (81–189) Intervention 83 ± 29.8 (45–136)	116 81	0.009 (10.626–66.101)
	BMI	9/13	Normal 96 ± 36.4 (45–154) Overweight 106 ± 37.0 (49–189)	95 107	0.544 (-43.072-23.414)
	Height	12/10	< 171 cm 110 ± 42.0 (49–189) ≥171 cm 93 ± 29.9 (45–136)	114 97	0.279 (-14.987-49.320)
	Surgeon	11/11	A 98 ± 33.1 (49–144) B 106 ± 40.3 (45–189)	95 106	0.608 (-41.037-24.673)
Reconstruction Time (Min.)	Device	11/11	Control 89 ± 11.1 (65–105) Intervention 87 ± 14.6 (62–110)	89 90	0.639 (-8.904-14.177)
	BMI	9/13	Normal 87 ± 8.4 (69–95) Overweight 89 ± 15.3 (62–110)	89 90	0.728 (-13.757-9.744)
	Height	12/10	< 171 cm 92 ± 11.6 (65–110) ≥171 cm 83 ± 12.8 (62–105)	92 84	0.104 (-1.989-19.789)
	Surgeon	11/11	A 87 ± 13.3 (62–110) B 89 ± 12.7 (65–105)	85 90	0.734 (-13.480-9.662)
Total Anesthesia Time (Min.)	Device	11/11	Control 259 ± 41.5 (140–284) Intervention 221 ± 41.0 (195–342)	259 221	0.046 (0.679–74.048)
	BMI	9/13	Normal 233 ± 45.1 (140–284) Overweight 245 ± 45.4 (184–342)	223 235	0.557 (-53.020-29.550)
	Height	12/10	< 171 cm 251 ± 42.1 (195–342) ≥171 cm 216 ± 42.4 (140–284)	256 226	0.190 (-13.643-64.410)
	Surgeon	11/11	A 235 ± 38.4 (184–295) B 245 ± 51.4 (140–342)	221 253	0.614 (-50.259-30.441)

Comparison of intraoperative time parameters between patients treated with conventional laparoscopic instruments (Control) and those treated with the TriSect rapide^®^ device (Intervention). Data are presented as mean ± standard deviation (SD), median, and range (min–max). Group comparisons were performed using the independent-samples t-test (two-tailed). The corresponding p-values and 95% confidence intervals (CI) for the mean differences are provided. Subgroup analyses were conducted according to body mass index (BMI), patient height, and operating surgeon. While total operative and anesthesia times were significantly shorter in the intervention group, mainly due to reduced preparation time, no significant differences were observed for reconstruction time or in any of the subgroup analyses

### Quality of life

**Fig. 4:**
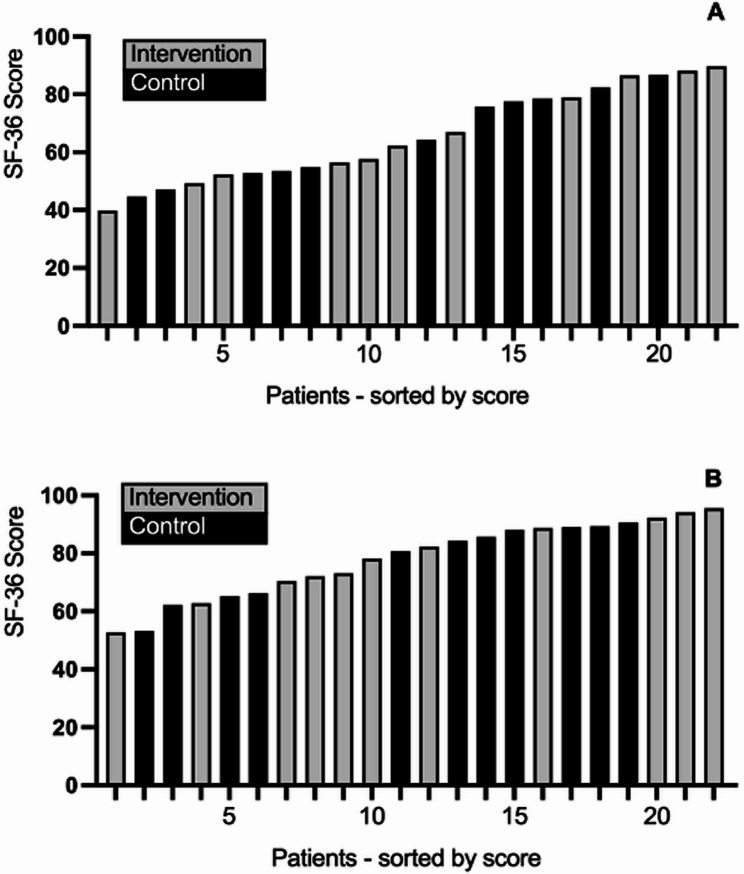
The 36-Item Short Form Survey Instrument (SF-36), sorted by value in a waterfall plot showed a uniform distribution of patients across the rating scale both pre-interventionally (**A**) and after the completion of treatment (**B**). In the SF-36, each item can score between 0 and 100, with a higher score indicating a better assessment of quality of life

All patients completed the SF-36 questionnaire. Values collected in the RAND SF-36 questionnaire are evenly distributed across the measurement scale for both time points: at the time of the initial consultation (preoperatively) and twelve weeks after the completion of treatment (Fig. 4).

No intergroup differences in the total SF-36 scores were observed at the time of the initial consultation, nor at twelve weeks after completion (intervention vs. control pre: mean 65.3 vs. 65.9, *p* = 0.8616, intervention vs. control post: mean 78.8 vs. 79.6, *p* = 0.7915). The level of subjectively perceived quality of life, as captured by the questionnaire, remained the same pre- and postoperatively (Table 4).

**Table 4:** Pre- and postoperative quality-of-life outcomes (SF-36)

SF-36 Domain	Preinterventional (Mean ± SD)			Postinterventional (Mean ± SD)		
	Intervention	Control	*p*-value (95% CI)	Intervention	Control	*p*-value (95% CI)
PF (Physical Functioning)	72.3 ±18.2	74.5 ±19.8	0.479 (-19.20-14.26	90.0 ±9.5	91.4 ±16.6	0.586 (-12.41-9.68)
RL (Role Limitations Physical)	56.8 ± 42.0	52.3 ± 30.5	0.750 (-28.29-37.38)	63.6 ± 46.6	65.9 ± 34.0	0.999 (-37.86-33.32)
BP (Bodily Pain)	69.3 ± 18.2	70.5 ± 23.4	0.910 (-19.84-17.57)	86.4 ± 15.3	84.8 ± 14.3	0.812 (-9.35-12.53)
GH (General Health)	66.8 ± 17.4	70.5 ± 15.4	0.750 (-18.25-10.97)	78.6 ± 15.5	77.7 ± 17.9	0.999 (-13.80-5.61)
VT (Vitality)	57.6 ± 44.9	60.6 ± 41.7	0.999 (-41.60-35.54)	63.6 ± 40.7	66.7 ± 33.3	0.750 (-35.41-29.35)
SF (Social Functioning)	63.6 ± 23.0	65.0 ± 22.7	0.500 (-21.70-18.97)	76.8 ± 20.0	75.5 ± 15.9	0.909 (-15.03-17.76)
RE (Role Limitations Emotional)	64.0 ± 19.8	66.2 ± 16.3	0.999 (-18.34-13.98)	76.7 ± 12.0	76.4 ± 11.9	0.999 (-12.21-12.94)
MH (Mental Health)	58.0 ± 21.8	56.8 ± 14.1	0.625 (-15.40-17.67)	78.4 ± 16.9	77.3 ± 16.6	0.999 (-15.43-17.70)
PCS (Physical Component Score)	67.7 ± 17.9	68.9 ± 18.6	0.511 (-17.43-15.05)	81.9 ± 14.3	82.6 ± 16.5	0.789 (-11.44-10.01)
MCS (Mental Component Score)	61.7 ± 20.8	63.3 ± 13.4	0.535 (-17.37-4.06)	74.2 ± 16.6	74.2 ± 10.9	0.906 (-13.07-13.13)
Total Score	65.3 ± 18.1	66.7 ± 16.0	0.373 (-16.58-13.83)	78.8 ± 13.7	79.2 ± 13.4	0.878 (-10.52-9.68)

Comparison of pre- and postoperative RAND SF-36 scores between the intervention and control groups. Data are presented as mean ± standard deviation (SD), p-values, and 95% confidence intervals of the mean difference. Statistical analysis was performed using independent two-sample t-tests after testing for homogeneity of variances with Levene’s test. No statistically significant differences were observed in any of the analyzed domains at either time point

**Fig. 5:**
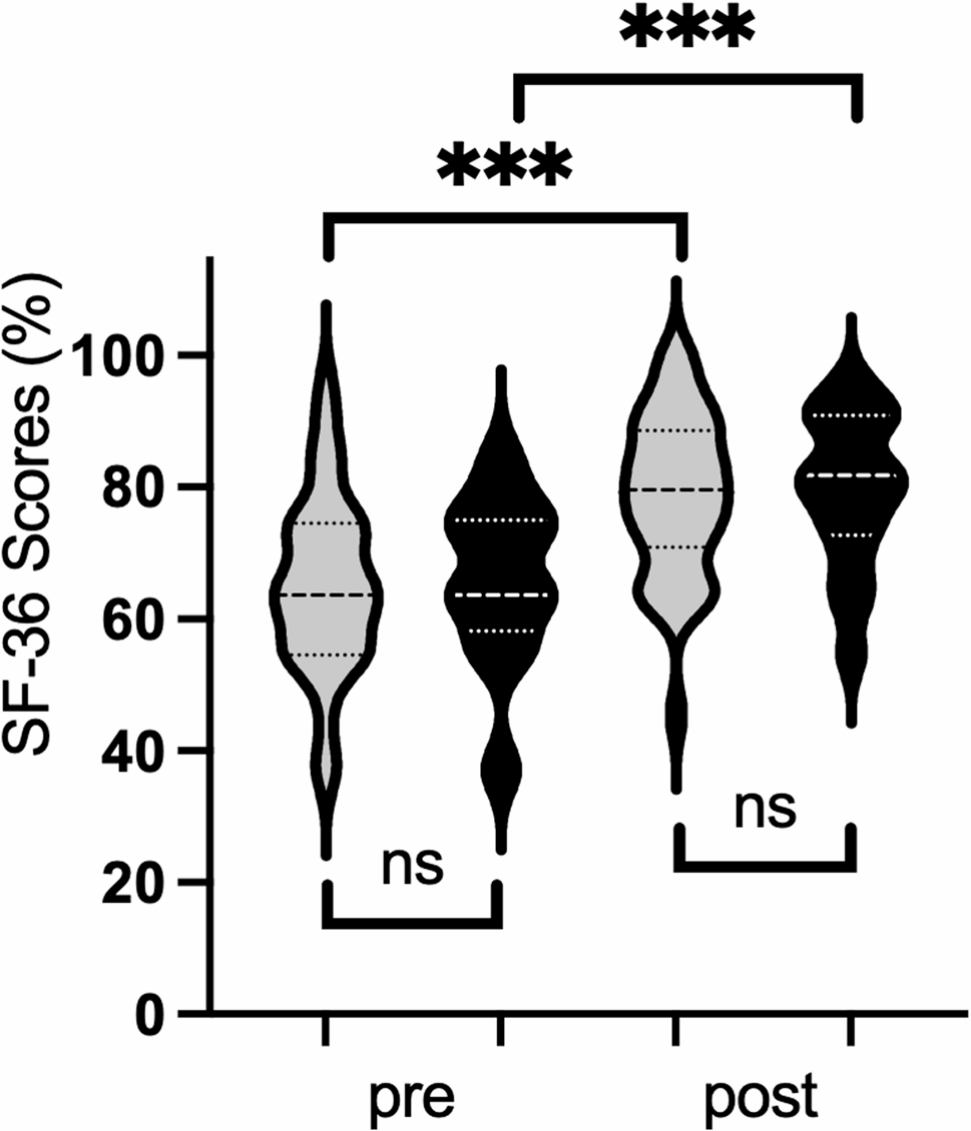
Distribution of the RAND SF-36 total scores in the intervention group (grey) and the control group (black) pre- and postoperatively. The means and 95% confidence intervals are presented as a violin plot. ‘ns’ indicates no significance, while ‘***’ indicates a *p*-value < 0.001

However, there was a difference in the assessment of personal quality of life in relation to the timing of the survey. In both groups, patients rated their quality of life at the time of the initial consultation significantly lower than twelve weeks after the completion of treatment (intervention pre vs. intervention post: mean 65.3 vs. 78.8, *p* < 0.001, control pre vs. control post: mean 65.9 vs. 79.6, *p* < 0.001) (Fig. 5).

## Discussion

This is the first randomized study to evaluate the TriSect rapide→ for laparoscopic pyeloplasty. The device demonstrated both safety and efficacy in tissue preparation, dissection, and cutting, without impairing wound healing. The primary study objective – demonstrating a reduction in operative time through the use of this multifunctional instrument – was achieved. A significant reduction in both total surgical time and the duration of the preparatory phase was observed in the intervention group using the multifunctional device. This effect is consistent with the expected advantages of the instrument, which was primarily utilized for dissection and hemostasis during the preparatory phase. In contrast, the reconstructive phase—mainly consisting of intracorporeal suturing—showed no relevant time reduction, as the device had only limited applicability in this part of the procedure. Subgroup analyses stratified by BMI, patient height, and surgeon did not reveal significant differences, further supporting the device’s specific impact on the preparatory part of the surgery. Likewise, we demonstrated no difference in postoperative quality of life or complications, both assessed as secondary endpoints.

With a median of 211 min in the control vs. 170 min in the interventional group, the operative times observed in the present study are consistent with the expected duration of surgery as reported in previous publications [20–22]. 

Previous studies in other surgical disciplines have similarly reported reductions in operative time associated with the use of multifunctional surgical devices. A meta-analysis focusing on adult populations demonstrated mean operative times ranging from 109 to 324 min [20]. In a randomized trial, Fagotti et al. demonstrated a significant reduction in operative time during hysterectomy [21]. Similarly, in a retrospective analysis, Hamamoto et al. reported a mean operative time reduction of 43.5 min when compared to the conventional approach, without an associated increase in complication rates. These findings are in line with the results of the present study, which likewise showed a comparable reduction in operative time during laparoscopic pyeloplasty [22]. Beyond these data, randomized trials in gynecology have shown that advanced bipolar vessel-sealing systems such as LigaSure can shorten or at least not prolong operative time compared with conventional techniques during vaginal hysterectomy and laparoscopic adnexal surgery, without higher complication rates [23, 24]. In addition, hybrid ultrasonic–bipolar technology (THUNDERBEAT) reduced operative time versus standard electrosurgery in a randomized study of radical hysterectomy, and in a randomized trial for neck dissection it lowered blood loss and shortened procedures without increasing complications [21, 25]. The TriSect rapide→ used in the present study employs tripolar/bipolar electrosurgery to enable grasping, dissection, sealing, and cutting within a single instrument. Despite differences in energy delivery compared with ultrasonic–bipolar hybrids, the shared mechanism—consolidating multiple steps and reducing instrument exchanges—likely explains the comparable reduction in operative time we observed during laparoscopic pyeloplasty.

The reduction in operative time observed in our study is not only a matter of procedural efficiency but also of clinical relevance. Several studies have demonstrated that longer operative duration in laparoscopic surgery is independently associated with higher postoperative morbidity and prolonged hospitalization. Jackson et al. reported that each additional hour of laparoscopic operative time increased the risk of complications [6]. Similarly, Catanzarite et al. found a significantly higher complication rate in minimally invasive gynecologic procedures exceeding 240 min [7]. A recent large cohort analysis by Unruh et al. confirmed that prolonged operative time during minimally invasive colorectal surgery correlated with longer hospital stay and higher complication rates [8]. In this context, the time savings achieved with multifunctional instruments such as the TriSect rapide^®^ are clinically meaningful, as they may help to reduce anesthesia exposure, surgeon fatigue, and perioperative risk, while maintaining the reconstructive precision required in laparoscopic pyeloplasty.

The introduction of a single-use instrument such as the TriSect rapide^®^ inevitably represents an additional cost factor that needs to be considered in the overall evaluation of surgical efficiency.

Although a detailed cost-effectiveness analysis was not part of the present study, it is reasonable to assume that shorter operative times and improved intraoperative handling could contribute to partial cost compensation. Comparable to previous health-economic evaluations on 3D versus 2D laparoscopy, a recent analysis reported mean per-procedure savings of approximately €437 in mid-level (Second-Level) hospitals due to reduced operating times and improved workflow efficiency [26]. While such savings may already offset a relevant portion of the additional instrument costs, a comprehensive cost analysis remains complex, as factors such as turnover times, staff utilization, and complication rates must also be considered [6, 27, 28]. Nevertheless, these findings suggest that the potential time savings associated with the TriSect rapide^®^ may, in the long term, translate into measurable economic benefits, particularly in surgical centers with similar case volumes and resource structures.

Given that the TriSect rapide→ operates on the basis of bipolar high-frequency energy, we considered the potentially thermal induced tissue affection during manipulation of human tissue. Morikawa et al., demonstrated in vivo that the use of various multifunctional instruments resulted in a local thermal spread ranging from 50.5 °C to 84.4 °C within a 1 mm radius of the target tissue [29]. For the dissector used in the present study, a thermal spread of less than 1 mm has also been proven in both, a porcine model and in vivo [30]. To date, no adverse intraoperative effects related to this phenomenon have been reported. In the present study, we were able to demonstrate that the application of the TriSect rapideⓇ in laparoscopic pyeloplasty is safe and does not result in an increased incidence of wound-healing disorders. The number of perioperative complications was comparable to those previously reported. Rassweiler et al., analyzed 189 patients an own cohort alongside with additional 412 patients from four different studies summarized in a meta-analysis focusing exclusively on complications associated with laparoscopic pyeloplasty and reported a rate of grade 3 complications ranging from 5.4% to 10% [31]. In some of the smaller cohorts, rates of up to 20% were observed [32, 33]. In our study, grade 3 complications were observed in 9% of cases in both groups. Of note, this corresponds to one case only in each group, and the small sample size limits the sense of percentage-based comparisons. In both cases, the complication consisted of an occlusion of the double-J stent, resulting in the formation of a perirenal urinoma. Management was replacing the stent; no further interventions, in particular no re-laparoscopy, were required. In fact, the most frequently observed complication in our cohort was urinary tract infection, occurring in three patients in each group. These infections required intravenous antibiotic therapy and are classified as grade 2 according to the Clavien-Dindo classification.

During the course of our study, Birkhäuser et al. published evidence from a prospective, randomized trial involving a cohort of 82 patients, demonstrating that double-J stent removal as early as two weeks after pyeloplasty is both safe and feasible. As these results were published toward the end of our recruitment phase, they could not be incorporated into our study protocol. However, they should be taken into account in the design of future prospective investigations [34]. 

In certain elderly or multimorbid patients, maintaining long-term ureteral stenting may represent an appropriate alternative to surgical reconstruction. During the screening phase, several such patients were initially stented but were excluded from the final analysis when a permanent drainage strategy was chosen. Within the analyzed cohort, all patients—including two older individuals aged 77 and 73 years—had a clearly confirmed ureteropelvic junction obstruction and opted for definitive surgical repair after thorough counseling regarding the long-term burden and procedural risks associated with repeated stent exchanges. Surgical reconstruction remains a reasonable treatment option even in elderly patients. In a study by Giri et al., approximately 20% of the investigated cohort were over 70 years of age, and the authors concluded that laparoscopic pyeloplasty can be safely and effectively performed in this population [35]. Nevertheless, a careful assessment of risks and benefits is essential, particularly considering that the hospital stay in elderly patients tends to be longer compared to younger individuals.

Assessing quality of life, we opted to use a more general questionnaire addressing the patients’ overall physical and mental condition. Although disease-specific instruments for urological conditions are available, to our knowledge, no validated questionnaire exists that specifically addresses patients with ureteropelvic junction obstruction. Given the primary interest in comparing patients’ subjectively perceived initial symptoms and the degree of symptom improvement following intervention, the validated and widely applicable SF-36 questionnaire was used in the present study. The domains addressing pain and physical functioning are central components of this instrument and are well suited capturing the course of recovery over time. In both groups, surgical intervention resulted in a significant improvement in quality of life compared to the preoperative status. Pre- and postoperative quality of life scores were comparable between the two groups. In the postoperative assessment, both groups reached quality of life scores—regarding both physical and mental health domains—that were comparable to those reported by Latas et al. [36]. The postoperative results are consistent with findings from two large-scale surveys conducted in healthy general populations. In a study by Jenkinson et al., a total of 13,000 healthy individuals in the United Kingdom were surveyed regarding quality of life [37]. The results obtained were comparable to those observed in the present analysis. Similarly, data from a German study involving 7,124 healthy participants also revealed quality of life scores that are in line with those reported in our cohort postoperatively [38]. 

During the procedures, all intraoperative fluids (blood, urine, and irrigation solution) were aspirated via the same suction system. As a result, it was impossible to accurately differentiate between the various types of intraperitoneal fluid. Consequently, the total amount of all collected fluids was documented, not blood loss only. No significant difference in hemoglobin levels was observed between the intervention and control group when comparing preoperative and immediate postoperative measurements. The observed decrease in hemoglobin levels —1.9 g/dL in the intervention group and 1.6 g/dL in the control group— is most likely attributable to hemodilution. This phenomenon is well known and has been described in a meta-analysis including a total of 2,794 patients, with a mean hemoglobin decrease of 1.55 g/dL was attributed to infusion-related hemodilution [39]. Given the average fluid volumes collected in the suction reservoir—150 mL in the intervention group and 280 mL in the control group—the observed hemoglobin drop cannot be explained by actual blood loss per se.

Multifunctional instruments are well established in laparoscopic urologic surgery and are continuously evolving. They enhance surgical performance by streamlining tissue handling, reducing instrument exchanges, and improving procedural efficiency. Based on the present data, the selected multifunctional instrument proved to be a valuable adjunct in the context of a complex reconstructive laparoscopic urological procedure, contributing meaningfully to operative workflow and time optimization. Given its versatility, this novel surgical instrument holds potential for broader applicability across various urological procedures, including kidney surgeries such as nephrectomies and nephron-sparing interventions, as well as prostatectomies — following a trajectory similar to that of previously established energy-based devices [40]. 

### Limitations

This study is subject to several limitations. The generalizability of the findings is restricted due to the small sample size and the single-center design. On the other hand, while the single-center design may limit external generalizability, it ensured a standardized surgical setting with consistent staff and perioperative management, thereby minimizing procedural variability. This homogeneity strengthens the internal validity of the study and allows for a clearer assessment of the effect of the new instrument. Long-term data were not collected, limiting conclusions regarding the sustained effectiveness of the intervention. Although quality of life was assessed using a validated questionnaire, it remains a subjective parameter. Furthermore, the study population was limited to patients from a specific region, and the technical equipment used may not be available in all clinical settings.

## Conclusion

The TriSect rapide^®^ is a safe and effective multifunctional instrument for laparoscopic management of ureteropelvic junction obstruction. Its integrated design facilitates tissue handling and dissection while reducing preparation and instrument exchange time, leading to a relevant reduction in operative duration. Without an associated increase in complications, these results suggest that the use of the TriSect rapide^®^ offers a measurable procedural benefit and may improve surgical efficiency in reconstructive laparoscopic urology.

## Data Availability

The datasets generated and/or analyzed during the current study are available from the corresponding author on reasonable request.

## Abbreviations

ASA: American association of anesthesiologists
BP: Bodily pain
BMI: Body mass index
CCI: Charlson comorbidity index
CT: Computed tomography
GH: General health
Hb: Hemoglobin
MCS: Mental component summary
MH: Mental health/emotional well-being
Min: Minutes
PCS: Physical component summary
PF: Physical functioning
QOL: Quality of life
RE: Role limitations due to emotional problems
RP: Role limitations due to physical problems
SD: Standard deviation
SF: Social functioning
UPJ: Ureteropelvic junction
URS: Ureterorenoscopy
VT: Energy/fatigue
WHO: World Health Organization
